# Comparative Symbiotic Effects of Mycorrhizal Fungal Strains from Different Hosts on Seed Germination and Seedling Growth in *Dendrobium officinale*

**DOI:** 10.3390/jof11100737

**Published:** 2025-10-14

**Authors:** Jian-Yu He, Xiao-Yan Xie, Zhuo-Qi Liang, Jian-Xia Zhang, Shu Liu, Xiao-Lan Zhao

**Affiliations:** 1 Guangdong Key Laboratory for Innovative Development and Utilization of Forest Plant Germplasm, South China Agricultural University, Guangzhou 510642, China; hjy88725@163.com (J.-Y.H.); sheyuhzu@163.com (X.-Y.X.); katherinepace@163.com (Z.-Q.L.); zhangjianxia@scau.edu.cn (J.-X.Z.); 2 Huajialing Forestry Station of Dingxi City, Dingxi 743305, China; 3 School of Life Science, Huizhou University, Huizhou 516007, China

**Keywords:** mycorrhizal fungi, host compatibility, *Dendrobium officinale*, seed germination, seedling growth

## Abstract

Compatible fungal partners of orchids can significantly enhance seed germination and increase seedling establishment under both in vitro and in situ conditions. This study isolated 14 *Tulasnella* isolates from five-year-old potted plants of three *D. officinale* cultivars. Three phylogenetically representative strains (Dca122, Dca222, and Dca113) and two additional orchid mycorrhizal fungus (OMFs, ML01 and Pi) were selected to evaluate their effects on *D. officinale* seed germination and seedling development in vitro, and subsequent seedling growth under greenhouse conditions. All five OMFs supported seed germination and seedling development in vitro. Notably, Dca113, Pi, and ML01 exhibited the most pronounced effects, producing protocorms 3–4 times larger in volume than controls. By day 25, 37.54%, 37.34%, and 42.6% of protocorms developed cotyledons with these isolates, respectively. Furthermore, after 120 days, ML01 and Dca113 treatments yielded 35.6% and 30.68% autotrophic seedlings with fully differentiated roots. Under greenhouse, ML01, Pi, and Dca122 significantly enhanced fresh weight accumulation, plant height, and stem node number in potted seedlings. In contrast, Dca222 primarily stimulated sprouting tillers and adventitious root formation. Our results demonstrate that the mycorrhizal effectiveness of OMFs from different hosts varies significantly in *D. officinale*. ML01 and Dca113 are ideal candidates for reintroduction programs due to their strong promotion of seed germination and rapid formation of rooted seedlings. ML01 proved the most effective OMF for enhancing growth in potted seedlings, while Dca222 demonstrated potential for co-inoculation strategies.

## 1. Introduction

Mycorrhiza is a specific symbiotic structure formed between plant roots and fungi. Mycorrhizal fungi can establish a symbiotic relationship with host plants and promote their growth [1]. Orchid seeds, lacking endosperm, rely entirely on fungal symbionts for carbon nutrients during early development, a phenomenon termed initial mycoheterotrophy [2]. The fungi that can promote seed germination are called germination-associated fungi. The fungi in Basidiomycota, which includes the Sebacinales, Tulasnellaceae, and Ceratobasidiaceae, represent the major group involved in the germination of orchid seeds [3,4]. There are currently two main methods for obtaining the symbiotic germination fungi critical for orchid seed germination. The first method is in situ symbiotic germination technology, which utilizes seed bags for germination in the original habitat. Once the seeds develop into protocorm or seedlings, the symbiotic fungi can be isolated from them, a process referred to as seed baiting [5]. The second method is tissue isolation, which primarily involves isolating endophytic fungi from the root segments or original protocorm of wild orchids. After fungal isolation, symbiotic germination experiments are designed to verify their seed germination-promoting effects [6]. In the symbiotic relationship between orchids and mycorrhizal fungi, one or more mycorrhizal fungi may facilitate seed germination and seedling development. However, some orchid species exhibit a strong specificity for certain mycorrhizal fungi [7].

Orchids exhibit complex symbiotic relationships with fungi throughout their lifespan, and the composition of orchid mycorrhizal fungi (OMFs) may vary at different developmental stages [2,8,9]. A notable example is the mycoheterotrophic orchid *Gastrodia elata*; the seeds rely on *Mycena* fungi for germination but require *Armillaria mellea* for subsequent growth [10]. In green terrestrial orchids, studies on *Cephalanthera* species [11] and on *Bletilla striata*, *Pecteilis radiata*, and *Spiranthes australis* [12] revealed that seedlings colonized by fungi exhibited a narrower phylogenetic range of fungal associates compared to germinating seeds, indicating a symbiotic fungal diversity bottleneck in protocorm stage. Similar diversity patterns were found in an epiphytic *Dendrobium moniliforme* [9,13]. These findings demonstrated that orchid mycorrhizal symbiosis exhibits a strong developmental stage specificity, and the protocorm stage represents a critical ecological filter. Moreover, the symbiosis may be further modulated by external factors such as phenological changes [14], soil microbial availability, and the cost–benefit stoichiometry of nutrient exchange [15]. Therefore, developing a systematic and context-explicit framework evaluating both orchid fungal functional partners and their environmental interactions is essential to leveraging the OMFs in reintroduction programs and commercial cultivation of orchids.

In previous studies, the assessment of symbiotic compatibility in orchids has often relied on a narrow set of metrics, such as germination rate or protocorm size, leading some orchid species to be broadly classified as generalists [16,17]. However, the generalization of specificity between orchid hosts and mycorrhizal fungi is increasingly questioned. For instance, Yamamoto [18] observed that the *Tulasnella* sp. strain HR1-1 isolated from *Pecteilis radiata* enhanced seed germination in *Bletilla striata* and produced pelotons, yet failed to increase protocorm biomass, indicative of partial compatibility between the fungus and host. In a more comprehensive approach, Fuji [12] evaluated symbiotic interactions across three terrestrial orchids (*Bletilla striata*, *Pecteilis radiata*, and *Spiranthes australis*) and five *Tulasnella* strains, including HR1-1 and four *T. calospora* isolates (MAFF305802–805) of Australian origin. It was demonstrated that the five *Tulasnella* strains had substantial varying effects on hosts, and only one *Tulasnella* strain supported development across all three phylogenetically divergent hosts.

*Dendrobium officinale* (Tiepi Shihu) is a critically endangered medicinal orchid endemic to East Asia. The Shi-Hu industry has developed rapidly in southern China since the 1990s as a result of massive commercial cultivation; however, due to doubts about the quality and efficacy of the products obtained with this cultivation method, massive commercial cultivation has not alleviated pressure on wild populations, according to recent surveys on the orchid trade in China [19]. Therefore, using OMF for restoration-friendly cultivation and population restoration protection is of great significance for the development of *D.officinale*. The fungal partners associated with *D. officinale* which enhanced seed germination, promoted nitrogen uptake, and stimulated polysaccharide accumulation included evolutionarily divergent fungal taxa (e.g., the basidiomycetous genera *Epulorhiza* and *Tulasnella* (Tulasnellaceae), alongside members of the order Sebacinales) [20,21,22,23]. However, most studies have focused on mycorrhizal fungi in adult orchids and during seed germination, leaving a critical knowledge gap regarding the seedling-associated mycorrhizal fungi (SAMF) [22]. The seeds rapidly germinated and formed protocorms by germination-promoting fungi. But, due to the lack of suitable SAMF, subsequent seedling development was often stunted or led to mortality. The OMF of *D. officinale* were generally obtained by in situ/ex situ baiting technique. However, in many native habitats, *D. officinale* has disappeared, so isolating mycorrhizal fungi from cultivated plants is an effective measure to protect wild *D. officinale.*

In the present study, we addressed four critical questions regarding mycorrhizal associations in *D. officinale*: (1) Do the fungal strains associated with the cultivated plants of the new varieties support seed germination? and if so, do these closely related isolates exhibit varying effects in promoting germination? (2) Do the fungal strains isolated from non-native orchids or non-orchid plants demonstrate similar promotive effects on *D. officinale* germination? (3) What are the distinct symbiotic effects exhibited by these germination-promoting fungal strains during seedling development? (4) What are the specific symbiotic effects of these fungal strains in enhancing seedling growth when applied under greenhouse conditions? These research priorities provide valuable insights for both the protection of natural habitats and the commercial cultivation of *D. officinale*.

## 2. Materials and Methods

### 2.1. Isolation and Identification of Endophytic Fungi in Dendrobium officinale Cultivation

Fresh roots were harvested from five-year-old potted plants of three D. officinale cultivars: ‘Zhongke No.1’, ‘Zhongke Congdu No. 2’, and ‘Zhongke No. 3’. These cultivars, known for their heat tolerance and disease resistance, were developed through successive generations of self-pollination from wild populations found in Guangnan County, Yunnan, and Dao County, Hunan [24]. Plants were cultivated at the Wanshan Research Centre, South China Agricultural University, Guangzhou, China.

The sampled roots were washed with running water for 1–2 h, surface-sterilized with 75% ethanol for 90 s and 0.1% mercuric chloride for 6 min, and then washed three times with distilled water. The root samples were blot-dried on sterile filter paper and were aseptically sectioned into 2–4 mm fragments using sterile scalpels. Fragments were plated on antibiotic-amended PDA (containing 1.5% streptomycin sulfate and 1.5% tetracycline hydrochloride) and incubated at 28 °C in darkness (Shanghai Yiheng Biochemical Incubator) until hyphal emergence. Hyphal tips were sub-cultured onto new PDA for purification. After repeating this purification step 4–5 times, purified strains were obtained. Colony morphology and mycelial characteristics were recorded after 5 days.

Among all the obtained fungal strains, those fungal strains in which the colony colors presented as pink, green, or black pigmentation were discarded. Remaining translucent-white isolates (*n* = 14) were preserved at 4–5 °C in the Guangdong Key Laboratory for Forest Plant Germplasm Innovation and Utilization for morphological studies on colony morphology and hyphal characteristics. Following DNA extraction using the CTAB method, the rDNA region, including the ITS1 (5′-TCCG TAGG TGAA CCTG CCG-3′) and ITS4 (5′-TCCT CCGC TTAT TGAT ATGC-3′) [25] primers, was amplified. The PCR procedure was as follows: pre-denaturation at 95 °C for 5 min; denaturation at 94 °C for 30 s; annealing at 54 °C for 30 s; extension at 72 °C for 1 min and 30 s, 35 cycles; and a final extension at 72 °C for 10 min. The PCR products were purified and commercially sequenced by Tsingke Biotechnology Co., Ltd. (Beijing, China). All ITS sequences obtained were blasted against the GenBank database (National Center for Biotechnology Information). Then, one sequence per species (from the isolate used below) was deposited in NCBI Genbank with an accession number (PV643361–PV643374).

### 2.2. Phylogenetic Analysis

We collected existing literature on Tulasnellaceae strains associated with *Dendrobium* species. The available ITS rDNA sequences of the Tulasnellaceae were downloaded from the GenBank database. A maximum likelihood (ML) phylogenetic tree was constructed based on the internal transcribed spacer (ITS) sequences of nuclear rDNA to elucidate the phylogenetic positions of the Tulasnellaceae fungi obtained in this study related to other known symbiotic fungi associated with *D. officinale* [20,21,22,26,27,28,29] (Supplementary Table S1), and representative functional Tulasnellaceae strains previously isolated from *Cymbidium* spp. within our research group [30,31,32,33]. All the sequences were first aligned using BioEdit v7.2.6.1, and then constructed phylogenetic trees using Mega11. Phylogenetic inference was rooted using the sequence *Serendipita indica* (syn. *Piriformospora indica*) as outgroup taxa to provide a reliable framework for interpreting evolutionary relationships within the ingroup. The *Serendipita indica* strain DSM 11827, which exhibits broad host compatibility, constitutes a distinct taxonomic unit [34]. Recent studies have confirmed that this generalist fungus established typical orchid mycorrhizal structures with diverse autotrophic orchids, enhanced host growth and nutrient acquisition [35].

### 2.3. Symbiotic Seed Germination Experiments

Three Tulasnellaceae fungal isolates (Dca113, Dca122, and Dca222) characterized in this study were used in symbiotic germination assays with *D. officinale* seeds. Additionally, *Tulasnella* sp. ML01, originally isolated from the roots of *Cymbidium sinense* [30], as well as the well-studied fungal endophyte *Serendipita indica* (syn. *Piriformospora indica*, strain DSM 11827), were included to assess comparative mycorrhizal efficiency.

*D. officinale* seeds were obtained via artificial pollination in April 2022 and harvested in November 2022. Immediately after harvest, the seeds were subjected to germination experiments. Seed disinfection involved treatment with 1% sodium hypochlorite for 5 min, followed by 5 rinses with sterile distilled water. The disinfected seeds were then suspended in 0.1% sterile agar solution. For germination assays, approximately 30–40 seeds (in 1 mL agar suspension) were transferred to Petri dishes containing 20 mL sterile oat meal agar (OMA; 4 g/L rolled oats, 8 g/L agar), overlaid with a nitrocellulose membrane. A 0.5 cm diameter mycelial plug was inoculated at the center of each Petri dish, while the control received a sterile agar plug. Eight replicate dishes per treatment were incubated in a growth chamber (JIUPO BPC500H) under controlled conditions with 25 ± 2 °C, 25 μmol·m^−2^·s^−1^ light intensity, and a 12 h light/dark photoperiod.

Seed germination, protocorm formation, and seedling development were assessed at 25 and 120 days post-incubation. Developmental stages were modified according to Arditti [36] protocorm formation (Stage 1), first leaf emergence (Stage 2), and second leaf development with sustained growth (Stage 3). To verify symbiotic establishment, representative protocorm materials from all treatments (sampled at 60 days) were processed for histology. Samples were embedded in 1.5 mL of 4% agarose and sectioned at 40 μm thickness using a Leica VT1000S vibratome (Leica Biosystems, Shanghai, China). Sections were mounted on glass slides and subjected to sequential staining, decolorization in 10% KOH (5 min, RT), neutralization with 1% HCl (4 min), staining with 0.05% trypan blue (3–5 min), and destaining in acetic acid-glycerol (10 min). Stained sections were examined under an Olympus BX51 compound microscope equipped with a Sony DSC-S85 digital camera.

### 2.4. Effects of Fungi on Seedling Growth Under Greenhouse

The uniform *D. officinale* seedlings (120-day-old, bearing 3–5 roots) pre-cultured on half-strength Murashige and Skoog (MS) medium were transferred to modified DE medium [32] and co-cultured with the five isolates (detailed in Section 2.3). After 30 days, the roots were sampled for verification of fungal colonization. Following confirmation of successful symbiosis (Supplementary Figure S1), inoculated and uninoculated control plantlets were transplanted into sterile pots (15 cm diameter) containing autoclaved substrate (pine bark: peat = 3:1, *v*/*v*). Each pot has 5 seedlings and there were 20 pots in each treatment. The initial growth parameters of experimental materials were recorded during transplantation, including the fresh weight, height, and root number. One day after transplantation, 10 mL of fungal inoculant (or sterile distilled water for controls) was applied to the rhizosphere. This inoculation procedure was repeated weekly, with a total of three supplemental inoculations. All plants were cultivated in greenhouse under partial shading (40% full sunlight intensity achieved via black shade cloth) with regular irrigation, and Hyponex No.1 (Sinochem Group, Beijing, China) at 1:1000 dilution was applied at two-week intervals.

### 2.5. Data Collection

After five months of cultivation, a minimum of 30 plants per treatment group were sampled, the growth parameters including fresh weight, plant height, node number, root number and length, and axillary buds were measured and calculated. For chlorophyll quantification, three to five mature leaves with uniform size from five randomly selected plants were sampled, rinsed with deionized water, and gently dried with filter paper. After removing the petioles and midribs, the leaves were cut, mixed thoroughly, and 0.2 g tissues were sampled for further analysis. Total chlorophyll contents were determined spectrophotometrically following the protocol of Li [37].

### 2.6. Statistical Analysis

The effects of different fungi on seed germination, seedling development and growth in *D. officinale* were compared with one-way ANOVA and Tukey HSD post hoc test method. All statistical analyses were performed using SPSS software (version 26.0).

## 3. Results

### 3.1. Fungal Isolation and Identification

In total, 14 fungal isolates were successfully purified from the roots of five-year-old potted plants from three *D. officinale* cultivars. All isolates were morphologically and molecularly identified as *Tulasnella* spp. (Table 1). After 7 days of cultivation, distinct morphological variations were observed among the isolates: the culture of Dca111 exhibited slower growth, with buff-colored basal hyphae; Dca113 demonstrated rapid growth (7.4 cm), forming circular, thin colonies with white, floccose aerial hyphae; Dca122 and Dca222 also displayed vigorous growth (6.8 cm and 7.0 cm, respectively), producing concentric colonies with distinct zones—inner buff-colored, densely immersed hyphae and outer white, sparsely distributed hyphae. All isolates adhered tightly to the medium without sporulation, and the hyphae exhibited septate branching at near-right angles, occasionally forming monilioid cells, consistent with the diagnostic features of *Tulasnella* spp. (see Supplementary Figure S1).

### 3.2. Phylogenetic Analysis

Thirty-one OMFs associated with *D. officinale* and *Cymbidium* spp., 14 isolates from this study and the strain DSM 11,827 were included in the phylogenetic analysis. Among the thirty-one OMFs strains, three (C2Y1, HCL3 and ML01) exclusively obtained from the roots of *Cymbidium* spp. formed typical peloton structures within host roots, and significantly enhanced host seedling growth and mineral nutrient absorption [32,33,42,43]. The remaining 28 OMFs were isolated from *D. officinale*, including 22 root-derived strains across multiple geographical regions and 6 strains obtained from protocorms (Table S1). All 28 strains demonstrated capacities to promote seed germination and/or seedling development in their native hosts.

**Figure 1:**
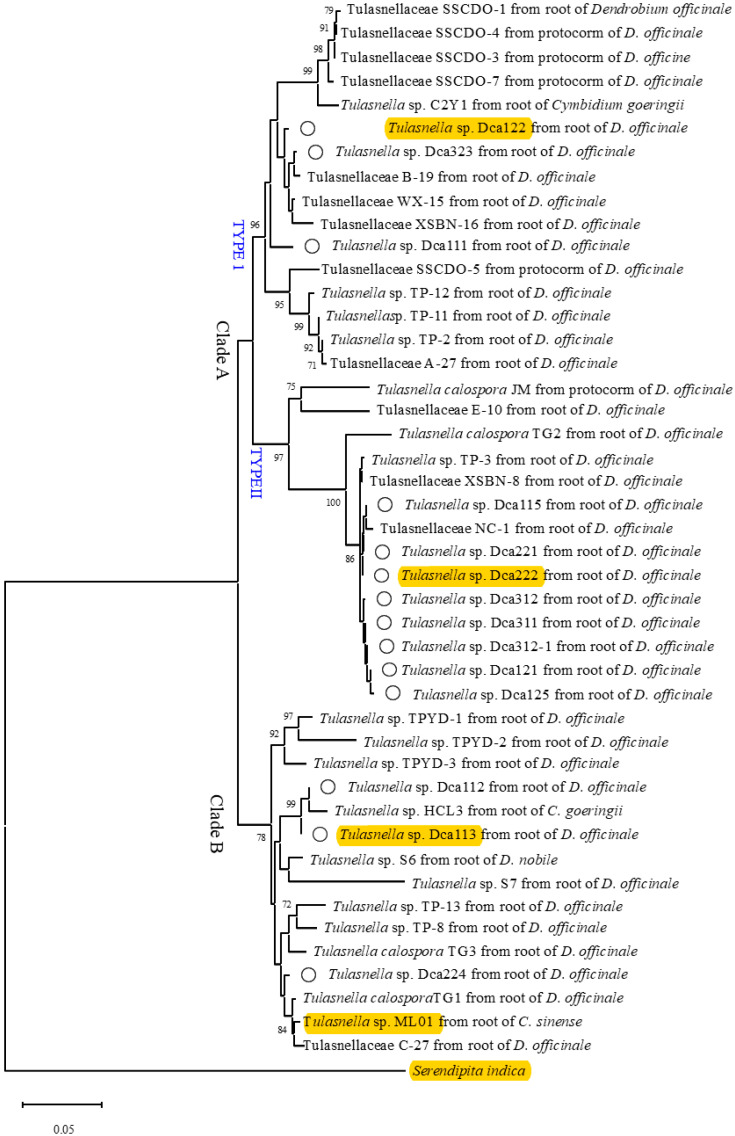
Maximum likelihood phylogeny of 45 *Tulasnella* strains associated with protocorms and adult plants in *D. Officinale* or *Cymbidium* spp. based on ITS-rDNA sequences. Bootstrap values (calculated from 1000 re-samplings) > 70% are shown at branches. *Serendipita indica* was the outgroup. Strains marked with a circle represent strains isolated from roots of *D*. *officinale* in this experiment. The isolates highlighted in yellow represent the *Tulasnella* strains selected for co-inoculation trials.

Phylogenetic analysis revealed that these 45 isolates clustered into two clades (Clade A and Clade B) with strong bootstrap support (Figure 1). Of these, 30 isolates clustered within Clade A, while the remaining 15 formed Clade B. Notably, Clade A was further differentiated into two well-supported subclades, referred to here as Type I and Type II. Among the 14 Tulasnella isolates recovered from the roots of *D. officinale* adult plants in this study, three (Dca122, Dca323 and Dca111) were clustered in Type I of Clade A, along with five isolates obtained from *D. officinale* protocorms and seven isolates from roots of *D. officinale*, as well as one isolate from the roots of *C. goeringii*; eight (Dca221, Dca222, Dca312, Dca311, Dca312-1, Dca121, Dca125, Dca115) grouped into Type II of Clade A, and the remaining three isolates (Dca113, Dca112, Dca224) fell within Clade B (Figure 1). Within Clade B, strains TP-8 and TP-13 were identified as core mycobionts for *D. officinale* seedling development [22]; strain TPYD-2, isolated via in situ seedling baiting, significantly enhanced root and leaf growth, plant height, and fresh/dry biomass accumulation in *D. officinale* seedlings upon inoculation [27]. Notably, strain ML01 exhibited broad host compatibility, serving as a predominant mycorrhizal partner across multiple *Cymbidium* species [30,32,33].

### 3.3. Effectiveness of Different Isolates on Seed Germination and Seedling Development in D. officinale

**Figure 2:**
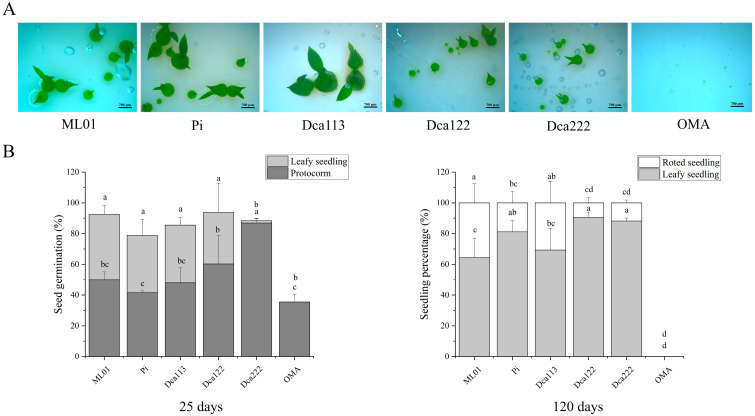
Four *Tulasnella* isolates (Dca113, Dca122, Dca222, ML01) and *Serendipita indica* (Pi) are compatible with *D. officinale* during the process of seed germination and protocorm development. (**A**) Morphological observations of *D. officinale* seed germination and protocorm developmental stages across all treatment groups at 50 days. Bars: 750 μm. (**B**) The percentages of seed germination, protocorm formation and seedling development (mean ± SE) in five fungal treatments and control treatment OMA of at 25 and 120 days after inoculation. Different letters next to error bars indicate statistically significant differences at *p* ≤ 0.05 (ANOVA with Tukey HSD post hoc test).

The results demonstrated that seeds inoculated with ML01, Pi, Dca113, Dca122, and Dca222 successfully progressed to the seedling stage, whereas the germinated seeds in the control group only reached the protocorm formation stage (Figure 2). At 25 days after inoculation, all fungal strains significantly enhanced seed germination compared to the control (*p* < 0.05), with no statistically significant differences observed among the different strains. However, their effects on protocorm development showed significant variations (*p* < 0.01) (Figure 2A,B). As shown in Figure 2A, the strains Dca113, Pi, and ML01 demonstrated the most pronounced promotive effects, producing protocorms 3–4 times larger in volume than controls (Figure 2A), with 37.54%, 37.34% and 42.6% developing cotyledons, respectively. In comparison, Dca122 resulted in inconsistent protocorm growth: approximately one-third of the protocorms were well-developed and bore cotyledons, while the remaining two-thirds were notably smaller. Dca222-treated protocorms exhibited significantly reduced size compared to the other strains (*p* < 0.01), showing minimal cotyledon formation (1.5%). In contrast, only 35.5% of the germinated seeds in the control group developed into small protocorms (Figure 2B).

**Figure 3:**
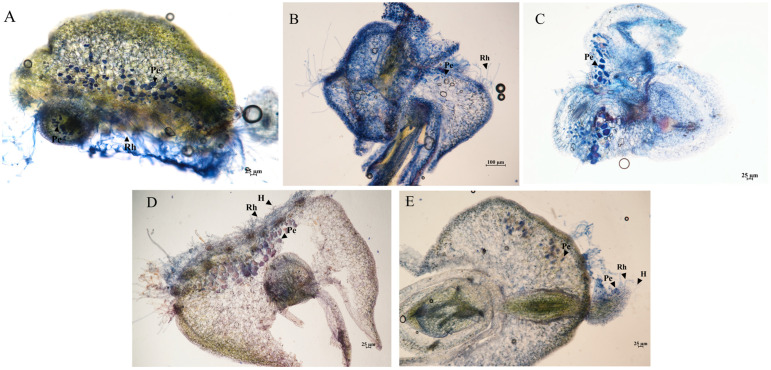
Trypan blue-stained longitudinal sections of symbiotically developing protocorms of *D. officinale.* (**A**–**E**): ML01, Pi, Dca113, Dca122 and Dca222. H, Fungal hyphae; Pe, pelotons; Rh, rhizoids. Scale bars: 25 μm (**A**,**C**–**E**); 100 μm (**B**).

After 120-day co-cultivation, protocorms on OMA medium remained arrested at the undifferentiated stage, whereas all fungal-treated protocorms developed into leaf-bearing seedlings (Figure 2B). Remarkably, the ML01 and Dca113 treatments produced 35.6% and 30.68% autotrophic seedlings with fully differentiated roots, respectively. The microscopic observation results showed that all fungal strains formed abundant fungal pelotons at the base of protocorms (Figure 3). These findings demonstrate that all tested fungal strains are mycorrhizal fungi of *D. officinale*, capable of establishing symbiotic associations with the host plant. Despite their shared mycorrhizal capacity, these isolates displayed significant functional divergence during the protocorm differentiation and development. Remarkably, strains ML01 and Dca113 emerged as the most effective microbial partners, demonstrating unparalleled capacity to promote: (i) protocorm transition to the seedling stage, and (ii) post-transition seedling development in *D. officinale*.

### 3.4. Comparative Symbiotic Effects of Different Fungal Isolates on D. officinale Seedling Growth in Pot

**Figure 4:**
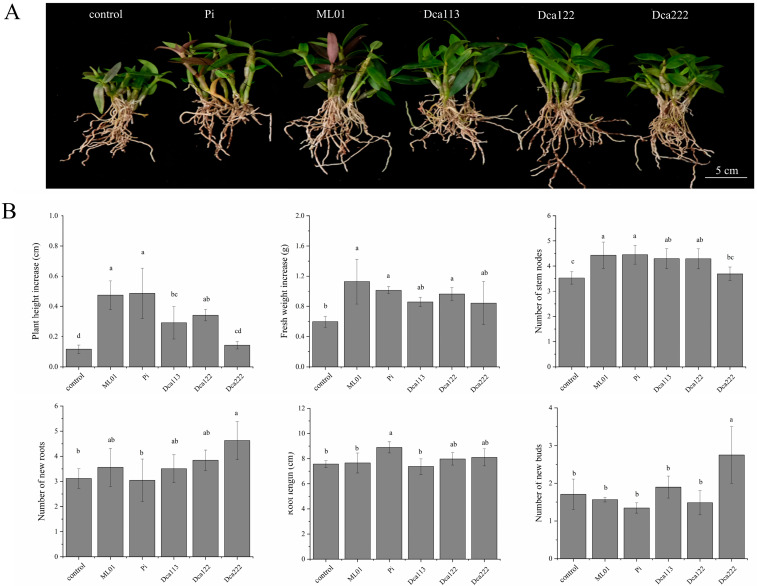
Comparative effects of fungal strains on seedling growth of *D. officinale*. (**A**) Fungal symbiont effects on *D. officinale* seedling growth after 6-month co-culture under greenhouse condition. Bars: 5 cm. (**B**) The effects of different synthetic fungal combinations on the growth parameters of *D. officinale*: plant height, fresh weight, stem nodes, root length, adventitious root formation, and tiller development (*n* = 45). Different letters next to error bars indicate statistically significant differences at *p* < 0.05 (ANOVA with Tukey HSD post hoc test).

To elucidate stage-specific symbiotic effects, we performed comparative assessment of five fungal associates (Dca113, Dca122, Dca222, ML01, and Pi) with *D. officinale* under controlled greenhouse conditions. Following 30-day co-culture on DE medium with six-month-old seedlings, light microscopy revealed that all fungal strains formed a symbiotic association with *D. officinale* seedlings, as evidenced by the presence of pelotons in the velamen and/or cortical region of the roots (Supplementary Figure S2). After five additional months of cultivation, three fungal strains (ML01, Pi, and Dca122) significantly enhanced fresh weight accumulation, plant height, and stem node number in *D. officinale* seedlings. Notably, only Pi demonstrated additional promotion of root elongation. Dca222 exhibited unique symbiotic effects, primarily stimulating sprouting tillers and adventitious root formation, consequently maintaining comparable plant height and node numbers relative to controls (Figure 4).

### 3.5. Strain-Specific Regulation of Chlorophyll Biosynthesis in D. officinale

**Figure 5:**
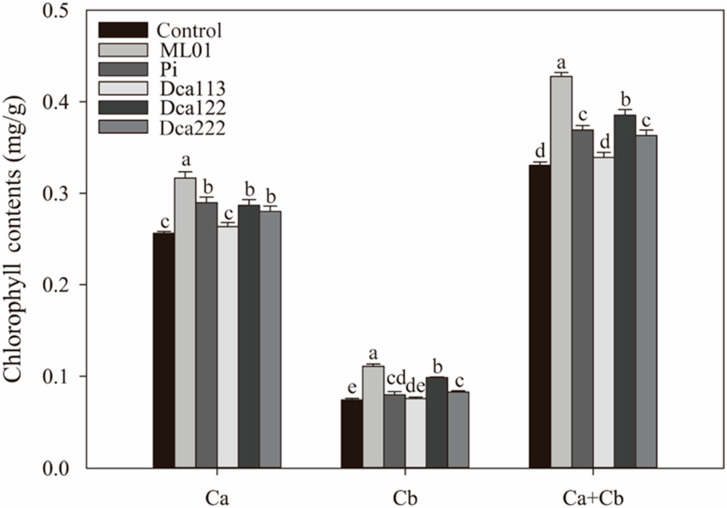
Comparative effects of fungal strains on chlorophyll biosynthesis of *D. officinale*. In each panel, different letters indicate significant differences (*p* < 0.05) based on the ANOVA with Tukey HSD post hoc test.

To investigate the symbiotic impact on chlorophyll accumulation in potted *D. officinale* seedlings, we compared the chlorophyll a, b, and total chlorophyll content of all the treated groups (Figure 5). Chlorophyll analysis revealed that among the five tested isolates, ML01, Pi, Dca122, and Dca222 significantly enhanced chlorophyll biosynthesis in potted *D*. *officinale* seedlings (*p* < 0.05), with ML01 eliciting the most pronounced effect—a 30% increase in total chlorophyll content. In contrast, Dca113 exhibited no significant influence on either chlorophyll *a*, chlorophyll *b*, or total chlorophyll levels compared to the control group (*p* < 0.05), indicating strain-specific modulation of chlorophyll biosynthesis.

## 4. Discussion

### 4.1. Cultivated Varieties Retain the Wild-Type Host Preference

Orchids (Orchidaceae) are dependent on mycorrhizal fungi for germination and to a varying degree as adult plants. Thus, utilizing OMFs to facilitate orchid seed germination and subsequent seedling growth could be an effective strategy for the conservation and artificial cultivation of endangered orchids [44,45,46]. To obtain and use optimal sources of fungal mycobionts, most related studies focused on isolating OMFs from the roots of adult plants or protocorms/seedlings in in situ trap experiments use and test their abilities to promote seed germination and seedling development in vitro and in the natural habitat [27,47]. However, a few studies have examined the recruitment of mycorrhizal fungi by cultivated orchid varieties.

In this study, 14 OMF isolates isolated from roots of artificially selected *D. officinale* cultivars were phylogenetically classified within *Tulasnellaceae* based on ITS sequencing. This family constitutes a major mycorrhizal partner for orchids, with numerous species supporting *Dendrobium* germination. Phylogenetic analysis revealed eight *Tulasnella* isolates (Dca221, Dca222, Dca312, Dca311, Dca312-1, Dca121, Dca125, Dca115) clustered within Type II of Clade A, showing close affinity to *Tulasnella* sp. NC-1 (MW432192.1), a strain from wild *D. officinale* in Jiangxi, China, confirmed to promote germination [28]. Three *Tulasnella* isolates (Dca122, Dca323, Dca111) clustered within Type I of Clade A in the phylogenetic analysis. They grouped with 12 other fungal strains obtained from adult roots or protocorms of *D. officinale*. Additionally, one of these strains C2Y1 was isolated from the root of *Cymbidium* spp. in our previous study [42]. Three isolates (Dca113, Dca112, Dca224) clustered within Clade B with 12 strains from wild *D. officinale* and *Cymbidium* spp. The phylogenetic positions of all cultivated–derived strains extensively overlapped with those from wild *D. officinale* and *Cymbidium* hosts, indicating unaltered fungal partner specificity under cultivation. Critically, the mycorrhizal composition of cultivated varieties (exclusively Tulasnellaceae) mirrors that of wild counterparts. It is worth noting that the fungi obtained in the current study may not represent the whole OMF community, and procedures to isolate the fungi could affect the fungi isolated.

### 4.2. Functional Differentiation Across Developmental Stages in Symbiotic Fungi

In this study, the results of symbiotic germination revealed that *D. officinale* is compatible with all the five fungal strains for seed germination (Figure 2) and protocorm form, although the efficiency of the strains differs. After 25 days of the symbiotic germination, strains of Dca113 and ML01 clustered within Clade B and Pi significantly promote the enlargement of protocorms compared to the control, but they do not significantly promote the formation of protocorms. In contrast, strain Dca222 showed an opposing promotive effect. This finding suggests a non-specific fungal association during germination in *D. officinale*. The low level of specialization during the germination phase is thought to reflect a lack of specificity in the initial signaling exchanges. Previous studies have demonstrated that compatible fungi for promoting seed germination may not be able to support subsequent seedling development [44]. The post-germination stage (protocorms to seedling development) must depend on a greater physiological compatibility with their respective mycorrhizal fungal partners. In the present study, all fungal treatments supported complete seedling establishment after 120 days of post-inoculation, showing no apparent stage-dependent fungal preference. Notably, ML01 and Dca113 treatments resulted in 35.6% and 30.68% of autotrophic seedlings with fully developed roots, respectively.

However, strains Dca222 and Dca122 significantly enhanced leaf formation (Stage 3) in symbiotic seedlings (*p* < 0.05), but exhibited markedly reduced root development capability compared to strains ML01 and Dca113 (*p* < 0.05). Under pot culture conditions, isolates ML01, Pi, Dca122 and Dca113 showed optimal increases in plant height, fresh weight, and the number of stem nodes. It demonstrated a similar growth-promoting effect on seedlings to that of *Tulasnella* sp. TP-8 (MN918482.1), *Tulasnella* sp. TP-13(MN918487.1) [22] and Tulasnella sp. TPYD-1 (MN545675.1), TPYD-2(MN545849.1), TPYD-3 (MN545858.1) [27]. In contrast, inoculation with strain Dca222 significantly enhanced new roots and bud formation, but had minimal impact on plant height or nodal development (Figure 4). The changes in plant growth pattern after inoculation with different fungi may be attributed to hormonal compounds derived from mycorrhizal fugus. These plant hormones, generated by symbiotic fungi, can influence the growth and development of host plants [20,48]. The symbiosis between *Cymbidum goeringii* and a Rhizoctonia-like mycorrhizal fungi causes the release of hormones, which can promote the growth of *C. goeringii* seedlings [49]. Previous studies have shown that plants generally produce multiple strigolactone (SL) species which are released from the roots and induce germination in seeds of the Striga and Phelipanche species in the soil [50]. SLs or their biosynthetic precursors act as plant hormones to inhibit shoot branching in plants [50,51]. Further research on plant hormones produced by mycorrhizal fungi could provide insights into the growth and development of *D. officinale* seedlings in symbiotic cultures. Moreover, given the demonstrated growth stimulatory properties of mycorrhizal fungi on *D. officinale*, co-inoculation with either ML01 + Dca222 or Dca113 + Dca222 may hold greater agronomic potential for enhancing plant development. Nevertheless, this synergistic hypothesis requires empirical validation through controlled trials.

### 4.3. Mycorrhizal Fungi Shared Among Orchid Species That Are Distantly Related

The fungi derived from the roots of *D. officinale* are not always more effective for seed germination and seedling development compared to those isolated from other orchid species. In the present study, ML01, isolated from the roots of wild *Cymbidium* spp. [30], and *P. indica*, sourced from the soil surrounding the roots of shrubs in the deserts of northwest India [34,52], exhibited similar promoting effects on the germination of *D. officinale* seeds and the development of protocorms compared to Dca113. Moreover, ML01 and Dca113 demonstrated a significant ability to enhance the growth of seedling roots compared to other fungal treatments. The *Epulorhiza repens* isolate ML01 formed typical OM symbiosis with *C. hybridum*, positively affected P, K, Ca, and Mg content in shoots and Zn content in roots of the plantlets and successfully established in pot orchids under greenhouse conditions [32]. *S. indica* (syn. *P. indica*), a fungus from the Serendipitaceae [53], is able to colonize a large range of hosts [54] as an endophyte, i.e., via loose colonization without symptoms. Recently, Xu [35] also confirmed that *P. indica* forms typical OM symbiosis and promotes the development of protocorms into plantlets in orchids from the subfamily Epidendroideae and tribes Cymbidieae and Dendrobieae. These results demonstrate that fungal strains derived from diverse hosts—including non-Dendrobieae orchids and even non-orchid species—effectively promote both seed germination and protocorm development in *D*. *officinale*, aligning with the findings reported by Zhang [44].

The acquisition of compatible OM fungi is often limiting for the successful wild reintroduction and conservation of orchids. In this study, the fungal strain ML01 and Pi demonstrated high host compatibility. Enhancing both seed germination rates and seedling growth including the height and stem node number. These results highlight their potential for ex situ propagation and habitat restoration of endangered orchids. However, it cannot be determined whether this fungus can promote the germination of *Dendrobium officinale* in natural environments, as a wide variety of fungi can promote seed germination in vitro, but there is strong ecological specificity in their natural habitats [44]. Thus, the functions of these OMFs in supporting seed germination, seedling establishment, and wild population reintroduction require further validation through field trials, combined with metabolomics and transcriptomics analyses to clarify the molecular mechanisms by which the *Tulasnella* sp. ML01 promotes *D. officinale* seed germination and subsequent seedling growth [55].

## 5. Conclusions

Acquiring functional orchid mycorrhizal fungi (OMFs) for conservation programs traditionally relies on isolating symbionts from wild adult orchids. This approach faces dual constraints: (1) potential discrepancy between seedling and adult mycobionts, and (2) inaccessibility of wild materials for over-collected species like *D*. *officinale*. To overcome these limitations, we established a cultivated-variety baiting protocol targeting root-associated fungi in nursery-grown *D. officinale*. Fourteen OMFs isolated from artificially selected *D. officinale* cultivars were phylogenetically classified within Tulasnellaceae. *D*. *officinale* exhibited compatibility with all five fungal strains across key developmental stages—seed germination, protocorm formation, seedling establishment and seedling growth—despite varying symbiotic efficiencies. Notably, no apparent stage-dependent fungal preference was observed. The symbiotic effectiveness of the tested mycorrhizal strains on *D. officinale* was independent of their host origins. Notably, the strain ML01, isolated from the terrestrial orchid *C. sinense*, consistently exhibited the most beneficial symbiotic effects across multiple developmental stages of *D. officinale*, indicating its significant value for conservation and cultivation practices.

## Figures and Tables

**Table 1:** Molecular identification of fungal species characterized in this study.

Fungal Isolates Codes	GenBank Accession Numbers	Sequence Identity (%)	Closest Relatives	References
Dca111	PV643361	99	MT611027.1	Downing [38]
Dca112	PV643362	99	KX774358.1	Solís [39]
Dca113	PV643363	99	KX929166.1	Solís [39]
Dca115	PV643364	98	KT601562.1	Davis [40]
Dca125	PV643365	98	MN173015.1	Meng [13]
Dca121	PV643366	99	MN173015.1	Meng [13]
Dca122	PV643367	99	MN645108.1	Jason [38]
Dca221	PV643368	99	MN173015.1	Meng [13]
Dca222	PV643369	99	MN173015.1	Meng [13]
Dca224	PV643370	99	KC758962.1	Pereira [41]
Dca311	PV643371	98	KT601562.1	Davis [40]
Dca312-1	PV643372	99	MN173015.1	Meng [13]
Dca312	PV643373	99	MN173015.1	Meng [13]
Dca323	PV643374	98	KT601562.1	Davis [40]

## Data Availability

The original contributions presented in this study are included in the article/Supplementary Material. Further inquiries can be directed to the corresponding authors.

## Supplementary Materials

The following supporting information can be downloaded at: https://www.mdpi.com/article/10.3390/jof11100737/s1, Figure S1: Fungal colonies and mycelial morphology; Figure S2: After 30-day co-culture of different fungi with *Dendrobium officinale* roots in DE medium, the root segments were sectioned and stained; Table S1: 3 orchid mycorrhizal fungi isolated from *Cymbidium* spp. (OMFs; HCL3, ML01 and C2Y1) and 28 OMFs were isolated from *D. officinale*. References [20,21,22,26,27,28,29,31,42,43] are cited in the supplementary materials.
