# Supplementary material for: Comparative Symbiotic Effects of Mycorrhizal Fungal Strains from Different Hosts on Seed Germination and Seedling Growth in Dendrobium officinale
Source: J Fungi (Basel). 2025 Oct 14;11(10):737. doi: 10.3390/jof11100737 (PMC12564960; doi:10.3390/jof11100737)
Supplement: Supplementary file 1 [file jof-11-00737-s001.zip › jof-3864997-supplementary.pdf]

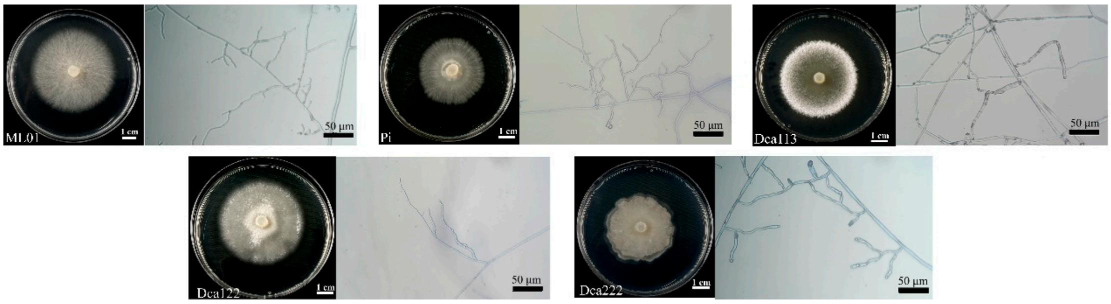

Figure S1 Fungal colonies and mycelial morphology

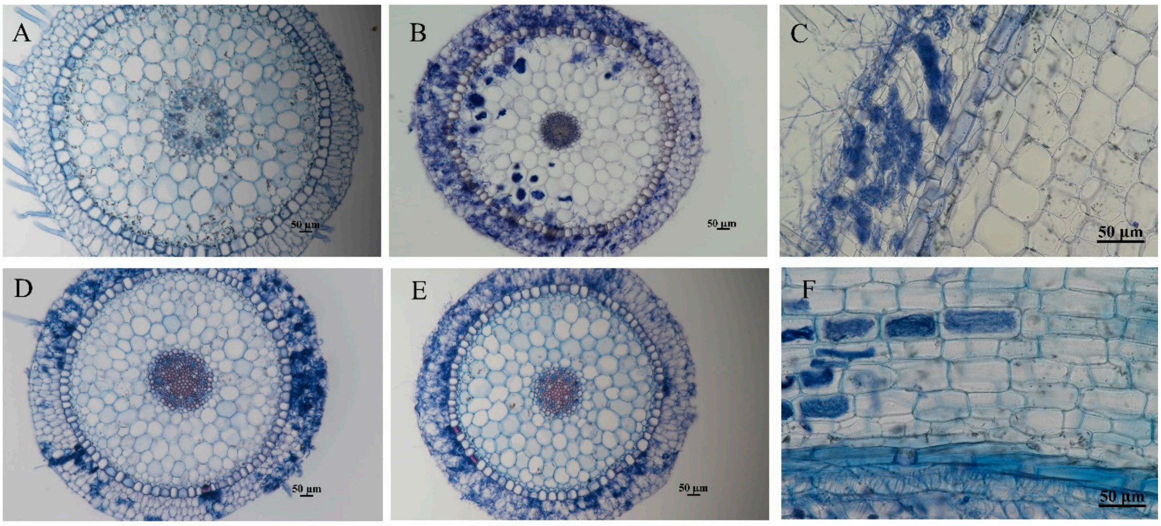

Figure S2 After 30-day co-culture of different fungi with *Dendrobium officinale* roots in DE medium, the root segments were sectioned and stained

A, Control; B, ML01; C, Pi; D, Dca113; E, Dca122; F, Dca222

Table S1 3 orchid mycorrhizal fungi isolated from *Cymbidium* spp. (OMFs; HCL3, ML01 and C2Y1) and 28 OMFs were isolated from *D. officinale*

Fungi species (GenBank accession No.)	Sampling sites	Species	Sources	Biological Effect	References
Tulasnella sp.HCL3(KJ499807.1)	Guangzhou	*Cymbidium goeringii*	roots	Promote seedling growth	[43]
Tulasnella sp.ML01(KJ499806.1)	Guangzhou	*C. sinense*	roots	Promote seedling growth	[31]
Tulasnella sp.C2Y1(KJ499809.1)	Guangzhou	*C. goeringii*	roots	Promote seedling growth	[42]
Tulasnella sp. TP-2(MN918476.1)	Yunnan	*Dendrobium officinale*	roots	Promote seedling growth	[22]
Tulasnella sp. TP-3(MN918477.1)	Sichuan				

Tulasnella sp. TP-8(MN918482.1)					
Tulasnella sp. TP-11(MN918485.1)	Yunnan				
Tulasnella sp. TP-12(MN918486.1)	Hunan				
Tulasnella sp. TP-13(MN918487.1)	Sichuan				
Tulasnella sp. TPYD-1 (MN545675.1)	Guizhou	*D. officinale*	roots	Promote seedling growth	[27]
Tulasnella sp. TPYD-3 (MN545858.1)					
Tulasnella sp. TPYD-2 (MN545849.1)	Guizhou	*D. officinale*	roots	Promote seed germination	[26]
Tulasnella calospora TG1(MN607233)	Chongqing	*D. officinale*	Roots or Protocorms	Promote seed germination	[21]
Tulasnella sp. TG2 (MN607234)					
Tulasnella calospora TG3 (MN607235)					
Tulasnella calospora JM (MN607232)					
Tulasnella sp. S6 (JN863900.1)	Yunnan	*D. officinale*	roots	Promote seed germination	[20]
Tulasnella sp. S7 (JN863901.1)	Yunnan		roots	Promote seed germination	
Tulasnellaceae A-27 (MW432188.1)	Guizhou	*D. officinale*	roots	Promote seed germination	[28]
Tulasnellaceae B-19 (MW432189.1)	Guizhou				
Tulasnellaceae C-27 (MW432190.1)	Yunnan				
Tulasnellaceae E-10 (MW432191.1)	Zhejiang				
Tulasnellaceae XSBN-8 (MW432193.1)	Yunnan				
Tulasnellaceae XSBN-16 (MW432194.1)					
Tulasnellaceae WX-15 (MW432199.1)	Anhui				
Tulasnellaceae NC-1 (MW432192.1)	Jiangxi				
Tulasnellaceae SSCDO-1 (MH34861.1）	Guangxi	*D. officinale*	protocorms	Promote seed germination	[29]

Tulasnellaceae SSCDO-3 (MH348612.1)
Tulasnellaceae SSCDO-4 (MH348613.1)
Tulasnellaceae SSCDO-5 (MH348614.1)
Tulasnellaceae SSCDO-7 (MH348616.1)
